# Current Sheet Antenna Array and 5G: Challenges, Recent Trends, Developments, and Future Directions

**DOI:** 10.3390/s22093329

**Published:** 2022-04-26

**Authors:** Sajjad Hussain, Shi-Wei Qu, Abu Bakar Sharif, Hassan Sani Abubakar, Xiao-Hua Wang, Muhammad Ali Imran, Qammer H. Abbasi

**Affiliations:** 1 School of Physics, University of Electronic Science and Technology of China, Chengdu 611731, China; sajjadhussain@uestc.edu.cn (S.H.); xhwang@uestc.edu.cn (X.-H.W.); 2 School of Electronic Science and Engineering, University of Electronic Science and Technology of China, Chengdu 611731, China; shiweiqu@uestc.edu.cn (S.-W.Q.); abubakarsharif@gcuf.edu.pk (A.B.S.); abkhassni@std.uestc.edu.cn (H.S.A.); 3 Department of Electrical Engineering, Government College University Faisalabad, Faisalabad 38000, Pakistan; 4 James Watt School of Engineering, University of Glasgow, Glasgow G12 8QQ, UK; muhammad.imran@glasgow.ac.uk

**Keywords:** antenna array, current sheet array (CSA), millimeter-wave, ultra-wideband (UWB), 5G

## Abstract

Designing an ultra-wideband array antenna for fifth generation (5G) is challenging for the antenna designing community because of the highly fragmented electromagnetic spectrum. To overcome bandwidth limitations, several millimeter-wave bands for 5G and beyond applications are considered; as a result, many antenna arrays have been proposed during the past decades. This paper aims to explore recent developments and techniques regarding a specific type of phased array antenna used in 5G applications, called current sheet array (CSA). CSA consists of capacitively coupled elements placed over a ground plane, with mutual coupling intentionally introduced in a controlled manner between the elements. CSA concept evolved and led to the realization of new array antennas with multiple octaves of bandwidth. In this review article, we provide a comprehensive overview of the existing works in this line of research. We analyze and discuss various aspects of the proposed array antennas with the wideband and wide-scan operation. Additionally, we discuss the significance of the phased array antenna in 5G communication. Moreover, we describe the current research challenges and future directions for CSA-based phased array antennas.

## 1. Introduction

In less than a decade, 5G has transformed from merely a distant concept to reality, with wireless access and services beginning to roll out across the globe. No doubt, 5G is not an evaluation but a big revolution, unlike predecessor generations. 5G mobile networks are an effective solution for the bandwidth scarcity led by the exponential growth of mobile devices and the ever-increasing demand for mobile applications [1,2]. In 5G, gigabits per second data rates are achieved by utilizing the unused millimeter-wave bands [3,4]. 5G technology will contain a range of networks to fulfill typical requirements of end-users such as voice, video, and data, along with the additional capabilities of seeming less connectivity across devices, machines, vehicles, sensors, and so on [5,6].

However, achieving the full potential of 5G requires end-to-end network transformations [7,8]. A phased array antenna is a crucial ingredient to unlock the true potential of 5G technology by attaining wider bandwidths and extended coverage, and better capacity at the millimeter-wave spectrum [9,10]. Although millimeter-wave systems can be relatively easily deployed for short-range indoor setups, many challenges are involved during their use for outdoor scenarios [11,12]. Many limitations should be resolved to realize millimeter-wave-based architecture, such as propagation loss, rain fades, atmospheric absorption, and high attenuation and shadowing [13,14]. 

Nonetheless, cell sites at millimeter-wave frequencies can cope with path loss, using antenna arrays with a large number of elements for steering multiple beams for superior coverage and capacity. Conventional base stations usually have between two to eight antennas, and this number can be in hundreds in 5G with antennas to form massive MIMO [15,16,17]. These electronically steered phased arrays enable signals steering/beamforming with greater precision.

Phased arrays for commercial deployment borrow beamforming and RF technology from arrays developed and deployed initially for military applications [18]. However, the recent advancement in semiconductor technology led to cost-effective solutions. Thus, phased arrays are now also used for commercial applications like satellites [19,20], radars [21], and 5G systems [22,23,24,25,26,27,28]. 

The main contributions of the paper are summarized as follows.

- Provide a comprehensive review of state-of-the-art antenna array designs based on the CSA approach for future wireless systems.
- Provide an in-depth insight into the existing designs and analyze the strength and weaknesses.
- Provide a discussion on the potential application areas in which these designs can be used.
- To highlight the significant research challenges that need to be addressed in the near future.

We hope that this survey will provide a foundation of knowledge on the topic and a way forward for further progress in this research area. The remaining paper is organized as follows. Section 2 briefly discusses the historical perspective and provides some fundamental concepts related to CSA-based phased array antenna technology and its deployment in 5G. It also assesses the challenges that are faced during the designing of high-performance UWB wide-scan phased arrays. Section 3 presents the review and categorization of proposed designs from different aspects. The paper ends with concluding remarks and future research directions.

## 2. 5G Communication and Role of CSA

5G communication networks will provide fixed as well as mobile broadband services to its end users. The 5G wireless interface, with low latency capability and ultra-reliable connections, will connect with many devices with diverse connectivity requirements to form the Internet of Things (IoT). Which includes industrial applications, utility networks, and advanced logistics.

### 2.1. Multi-Layer Frequency Spectrum Approach

A multi-layer frequency spectrum approach is necessary to fulfill such a wide range of user requirements and scenarios [29,30]. The 2 to 6 GHz spectrum provides the best compromise between coverage and capacity. The 26 GHz, 28 GHz, and 37 GHz bands facilitate users with the demand for high data rates. The utilization of spectrum below 2 GHz to provide deep indoor and wide-area coverage. This multi-layer frequency spectrum approach is further deliberated in Table 1. 

Due to the highly fragmented electromagnetic spectrum, antenna designers face challenges in developing solutions for unique frequency bands. The solution could be to consolidate several bands into a multifunctional aperture. Ultra-wideband (UWB) arrays can be used to accomplish this challenge. CSA is one of the array designs approaches that can provide antenna arrays with operational bandwidth of a decade and more. Therefore, such arrays can result in a single aperture with wideband capability for several band operations. Additionally, these arrays can reduce the integration cost and ease other system-level requirements.

**Figure 1:**
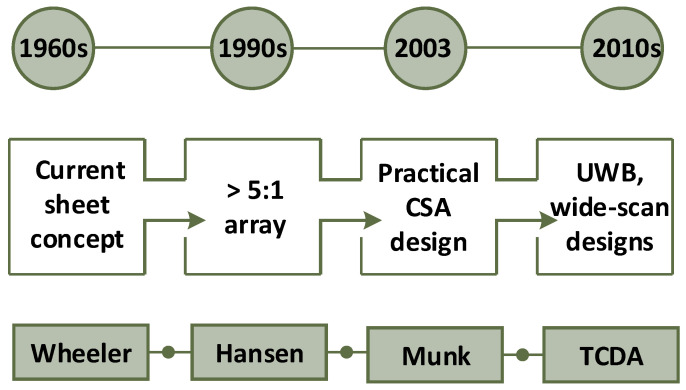
Historical perspective and present state of CSA technology.

Traditionally phased array antennas were developed for and deployed in military applications. In the recent past, low-cost semiconductor components used in microwave and millimeter-wave systems have been readily available and are cost-efficient, thus leading to the deployment of phased array designs for commercial applications. After the advent of the radar in the 1940s, there was continuous research to improve the scan capabilities of phased arrays. The current sheet was a theoretical concept proposed by Wheeler in the 1960s, which eventually led to the realization of CSA [31]. However, the first practical implemented CSA design was reported in 2003 [32]. In the last decade, many state-of-the-art UWB and wide-scan array designs based on this concept have been reported in the literature. The earliest phases and subsequent evolution of CSA technology are presented in Figure 1. 

### 2.2. CSA Design Approach

The CSA is a fundamentally different design approach from conventional methods to realize wideband arrays. CSA exhibits wider bandwidth when deployed as a frequency selective surface (FSS) [33]. This is due to the inherent capacitance between the closely spaced dipole elements. Mutual coupling is used as the main design parameter in CSA. The capacitive coupling between the array elements is used to counteract the destructive shunt inductance originating from the ground plane. In this way, the array input impedance is primarily real, even for low frequencies, with even minimal height above the ground plane. Moreover, the impedance variation due to scan angle can be curtailed using closely spaced array elements. This approach works very similarly to the theoretical ‘current sheet’ concept proposed by Wheeler, thus named CSA.

**Figure 2:**
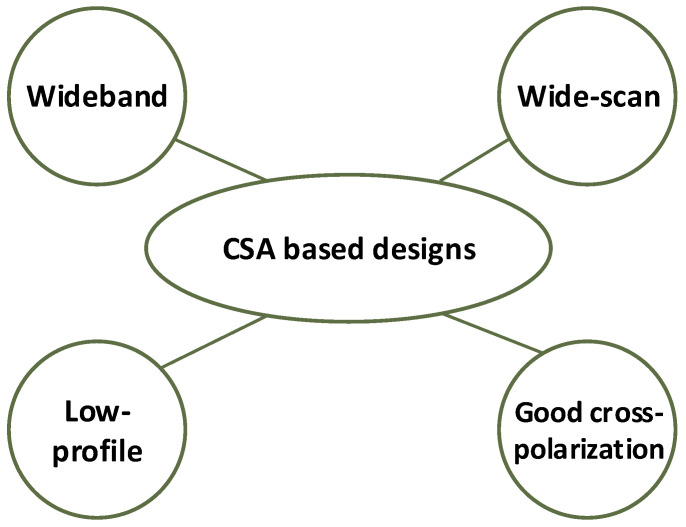
Advantages of CSA-based array design.

The benefits of inter-element coupling, low dielectric profile, and overcoming the resonant behavior, eradicate the sudden impedance variations and result in an array with UWB array design. The CSA unit elements are generally sized using the highest frequency, for instance, for a square array lattice with a size nearly half of the wavelength at the highest frequency or less. The small inter-element spacing eliminates the grating lobes within the desired band and offers better potential for the wide-angle scan. CSA also provides a low-profile aperture with a thickness generally on the order of λ/10 at the lowest frequency. However, the thickness at the lower frequency band is determined keeping in view the maximum tolerable impedance mismatch. These attributes make CSA a suitable choice for low-profile and conformal applications [34,35,36,37]. The polarization characteristics of a phased array are dependent upon the type of radiating element selected. CSA also provides orthogonal array configuration to design a dual-polarized array with superior cross-polarization isolation. To obtain dual polarization each CSA unit cell is comprised of one horizontally (*H*) polarized radiator and one vertically (*V*) polarized radiator. The phase centers for *V* and *H* polarization are not coincident. CSA is easy to operate in dual-polarization due to the simple antenna structure. Moreover, CSA brings superior performance, in terms of total electrical thickness and maximum scan angle. Some of the salient benefits of CSA-based antenna design are presented in Figure 2.

The CSA design principle features a periodic lattice to avoid grating lobes, an appropriate ground-plane spacing to overcome the half-wavelength radiation null (that can appear at the upper band end), a dielectric profile, and the coupling between the elements through wideband matching techniques. Some additional features need to be considered before practical implementation, these are the size of the finite array, feeding structure, and element coupling physical implementation. All mentioned features are critical in obtaining good performance.

**Figure 3:**
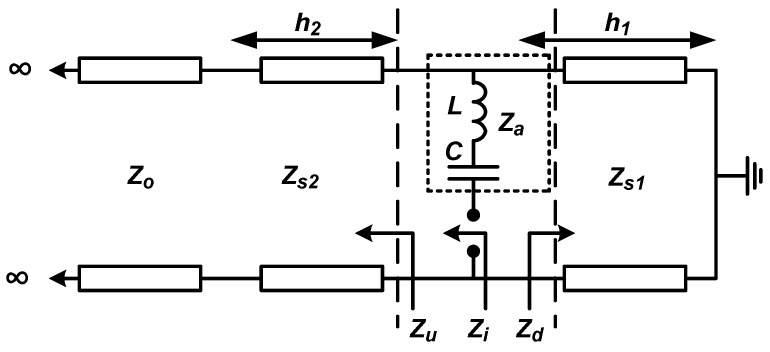
A typical TCDA equivalent circuit.

The famous tightly coupled dipole array (TCDA) [38,39,40,41,42,43] is based on the CSA design technique. The equivalent circuit of a typical planar TCDA unit element with a ground plane and a superstrate is presented in Figure 3. The antenna and the ground plane are separated by substrate with distance *h*_1_, and the antenna is loaded with superstrate with profile *h*_2_. Antenna impedance is composed of inductance (*L*) of dipoles and capacitance (*C*) due to coupling. The size of the spacing between nearby dipoles and the shape of the dipole antenna determine *Za*. However, the geometry of the array structure determines the embedding impedances *Zd* and *Zu*, which are impedances toward the inward and outward direction of the antenna, respectively. If the input impedance, *Zi* = *Za* + *Zu*/*Zd*, is matched over a wide range of frequencies, the TCDA can have a wideband operation.

### 2.3. Challenges to Design a High-Performance CSA

CSA provides wide impedance bandwidths and superior scanning features however, an antenna array with many elements is difficult and complex to design compared to an isolated antenna. The key challenges for high-performance CSA are:

- Efficient feeding with the wideband operation;
- Common mode resonance;
- Low active voltage standing wave ratio (VSWR);
- Avoiding grating lobes;
- Surface waves;
- Bulky parasitic substrate.

For wide-scan arrays, it is necessary to have a lossless feeding scheme for efficient radiation. However, to design such lossless impedance matching circuitry is challenging since it leads to resonant common modes, along the feedlines. Although the unbalance feed structure makes fabrication easier and facilities the scalability of the array to higher frequencies, common-mode resonance phenomena are frequently observed in such feed. Consequently, any type of feed can be used for input impedance matching, but suppression of common-mode resonance is critical to achieve wideband performance. 

When all elements of the array are excited simultaneously, the mutual coupling between the elements is also considered to evaluate the active VSWR. A low active reflection coefficient is required however difficult to achieve in a wide-scan, wideband phased array. The active reflection coefficient is given by

(1) $$Active\;S_{m}=\overset{N}{\underset{n=1}{\sum }}\frac{a_{n}}{a_{m}}S_{mn}\;,m=1,2,\ldots ,N$$

*N* represents the total number of ports, the excitations of *m* and *n* elements are given by *a_m_* and *a_n_*, respectively. These excitations are complex and constitute phase and magnitude. The transmission coefficient between the corresponding elements is represented by *S_mn_*. Then, the active VSWR of the desired array element is calculated by (1 + $Active\;S_{m}$)/(1 − $Active\;S_{m}$).

The transmission coefficient does not change during the array scanning. However, the complex excitations of the elements changes and this can cause impedance mismatch due to the high reflection coefficient. The mutual coupling between the elements can also cause scan blindness. Mutual coupling in CSA is useful; however, controlling and quantifying it is still somewhat tricky. If scan blindness occurs, it leads to lower efficiency since most of the power will be reflected.

Another common design challenge in CSA is to avoid the occurrence of grating lobes since the beam of this lobe has nearly the same gain as the main beam. If it occurs, it leads to lower gain and interference with the main beam. Element spacing needs to be set carefully to avoid the occurrence of grating lobes. Different requirements need to be achieved for rectangular and triangular grid arrays.

(2) $$D_{x},D_{y}<\frac{\lambda_{h}}{1+\left|\sin\left(\theta_{0max}\right)\right|}$$

(3) $$D_{x}<\frac{1}{\sin\alpha }\;\frac{\lambda_{h}}{1+\left|\sin\left(\theta_{0max}\right)\right|},\;\mathrm{subject}\;\mathrm{to}\;\frac{\pi }{6}<\alpha <\frac{\pi }{3}$$

(4) $$D_{y\;}<\frac{1}{\cos\alpha }\;\frac{\lambda_{h}}{1+\left|\sin\left(\theta_{0max}\right)\right|},\;\mathrm{subject}\;\mathrm{to}\;\frac{\pi }{6}<\alpha <\frac{\pi }{3}$$

*D_x_* and *D_y_* represent the element spacing in the *x* and *y* planes, respectively. In the elevation plane, the maximum scan angle is *θ*_0*max*_ and the basic angle for the isosceles triangle is *α* for the triangular grid. 

As evident from the above-mentioned formulae, the array element spacing is linked to the free space wavelength at the highest frequency. Therefore, to avoid the grating lobed appearance, the spacing of the elements needs to be set according to the mentioned guidelines.

In TCDA, superstrate loading above the aperture can significantly improve the scanning performance of the array. However, this sometimes results in the excitation of surface waves, particularly for large scan angles. Thus, solutions to avoid these surface waves are needed. Moreover, a bulky dielectric superstrate is not required as this can lead to cost and weight increases. Furthermore, this can significantly increase the thickness of the array, more lately, low-profile arrays are in high demand [44,45]. 

## 3. Recent Trends and Developments

The last decade has seen an increased demand for phased array antennas, particularly for commercial applications, mainly driven by 5G. The ever-increasing demand for wide-bandwidth communications for future wireless systems has led researchers to apply efforts to address these challenges and needs. However, a particular design technique can address only partial challenges. In the coming paragraphs of this section, CSA-based phased arrays with wideband and wide-scan angle characteristics are presented and discussed.

### 3.1. UWB Array Designs

The progress of antenna array designs with UWB, miniaturization, high-gain, and low-scattering features have attracted growing attention. Recently, several studies on UWB phased array designs have been reported, for instance. One of the first CSA-based array designs developed and validated through hardware measurements was [46] from Harris corporation back in 2007. This approach treated the aperture of the array as a periodic surface to ensure the performance of each element within the array environment. This was contrary to traditional array design based on isolated elements and did not make up for the mutual coupling amongst the elements when placed in an array environment. This design approach of tightly coupled elements set a pathway for array designs with bandwidths nearing a decade. Frequency selective surface (FSS) was incorporated in an array based on tightly coupled elements [47] to suppress the destructive interference by the ground plane. This resulted in an increase in the bandwidth of the array by a factor of more than two. Moreover, a superstrate is used to overcome the losses due to the FSS’s resistive nature. This successful integration of FSS made this array a low-profile (0.055*λ_low_*). The array achieved a 21:1 bandwidth (from 0.28–5.92 GHz) for the practical benchmark for UWB arrays of VSWR < 3.

A tightly coupled spiral array to achieve a 20:1 impedance bandwidth array aperture is reported [48]. To model, the radiation impedance of this wideband array of equivalent circuits is proposed, including the representation of superstrates and higher-order grating lobes. The inclusion of higher-order modes was critical as these modes can influence array impedance. Interwoven arms made it possible for adjacent elements to overlap. These arms overlapping played a crucial to achieve the wideband performance. A patch array based on tightly coupled elements with low-profile and small size is reported [49]. Interestingly FSS is used as a radiating structure, and this approach exploited the different behavior of FSS when operated in passive and radiating modes. The broadband operation is achieved using periodic excitation and coupling amongst elements. However, due to the inherent narrowband property of patch antennas, the array failed to achieve wideband operation and has an impedance bandwidth of 5.6% at 2.07 GHz. In Table 2, the reported UWB arrays are compared in terms of bandwidth, scanning range, unit element dimensions, and gain. Notably, dimensions of the unit element are mentioned in terms of *λ_h_*, i.e., wavelength at the highest operating frequency for a fair comparison. Moreover, all bandwidths are calculated under the criterion of active VSWR ≤ 3.

A novel technique based on characteristic modes for aperture excitation of finite element UWB arrays is reported [50]. Notably, the proposed taper provided wideband matching for all array elements, including the edge elements. This approach is unlike the traditional excitation tapers that are mainly used for beam shaping. However, this method relies on the arrays M × N mutual impedance matrix, which needs to be measured or precomputed. Since this technique caters to the good matching of edge elements, it is suitable for array designs based on CSA because edge element performance is significantly affected by the edge effect. This excitation approach assured superior performance and aperture efficiency in a planar array. Moreover, due to its simplicity, this method can easily be incorporated into the design process to optimize unit elements and finite array geometries.

The famous planar Ultrawideband phased array (PUMA) is also based on the CSA concept. Its first design was reported [41] almost a decade ago. The distinct feature of PUMA is the introduction of a novel feeding scheme that facilitated the fabrication of prototypes using standard PCB technology. Moreover, the fabrication of the shorting posts and feed lines are implemented as plated vias. Moreover, the new feed method eliminated the need for the external balun. This dual-polarized array design achieved 5:1 bandwidth, with good cross-polarization and wide-angle scan operation to 45° in all three planes. As mentioned in the previous section, common-mode resonance is catastrophic in CSA. PUMA design used shorting posts to overcome common-mode resonance. Another PUMA dual-polarized array with 3:1 bandwidth, unbalanced feed, and fabricated using standard multi-layer PCB technology is reported in [51]. The salient feature of this array aperture that constitutes tightly coupled dipoles is it’s low-profile (0.15*λ_low_*). Common-mode resonance was observed because of unbalanced feed and carted using a pair of plated vias connected to the ground, which moved the problematic resonance out of the band.

A low-profile UWB phased array antenna with integrated balun [52] proposed an alternative solution instead of a non-uniform characteristic mode excitation scheme [50], as just discussed above. The alternative solution made periphery elements short circuits and employed standard uniform excitation for central array elements. Notably, the proposed technique resulted in around 3 dB more gain and much higher efficiency than the commonly used resistive termination technique for medium-sized arrays. A compact 10:1 balun is integrated into the design to feed the array. This design provided a better solution than using matched loads for edge effect alleviation. Although the matched loads method improves the array bandwidth, this is done at the trade-off of realized gain and efficiency.

**Figure 4:**
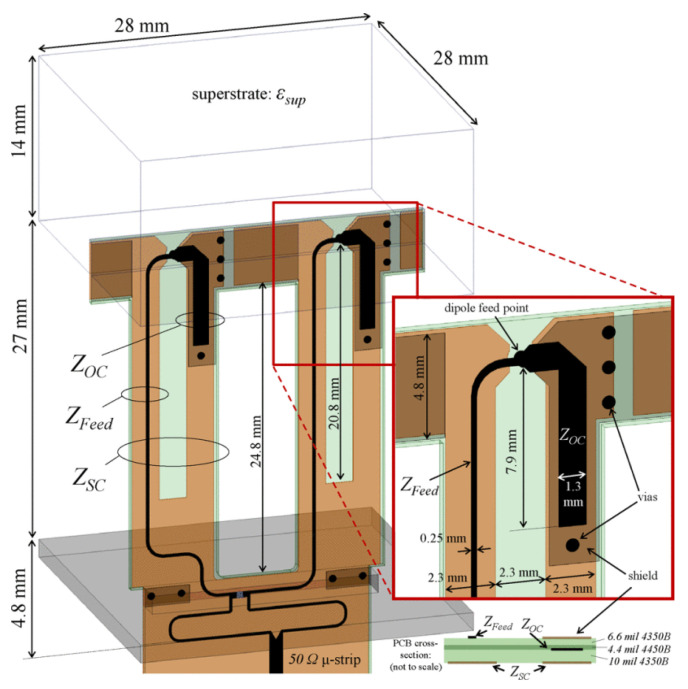
Unit element implemented with an integrated balun, each cell has two 100 Ω baluns fed using a single 50 Ω microstrip trace [38].

Interestingly, non-symmetric dipole arms with strong inter-element coupling are used as an aperture to design a planar array with X-band operation using a matching network with an integrated feed [53]. The array reported 60° and above scan performance in principal planes. The only drawback is the oversampled size of the element which is 0.34*λ_h_*. Moreover, the bandwidth of this array can be much larger, but the feed design limits it. As reported for the dual-polarized array with an integrated balun having more than 7:1 bandwidth used the reactance of a Marchand balun for impedance matching [38], reproduced in Figure 4. This feeding technique led to wideband, and since no external bulky balun was used, the size was reduced significantly. This solution successfully overcame the drawback of [53] in which the feed design was not equally wideband as the array.

A 4.5:1 array was designed using an equivalent circuit, which considers the representation of arrays based on dipole elements from DC to the first free-space grating lobe frequency [54]. This circuit model was also used to calculate the ground plane distance and optimal dielectric arrangement to improve scanning and bandwidth performance. Owed to the strong mutual coupling, an omnidirectional antenna array with a wideband 1-to-12 feed network is reported for 1.7–3.54 GHz [58].

Printed arc dipoles are placed to form a ring with overlapped edges to achieve strong mutual coupling. Two rows of parasitic arc strips are placed around the excited dipoles for impedance matching. An arc strip was used as a director for enhanced radiation in the horizontal plane. The reported design stacked to form a vertical array can be used for high gain base-station applications. Another array designed for satellite communication with 6:1 bandwidth from UHF to the lower-C band also used a similar inter-element coupling [55]. The latter employed a dual-offset feed for minimized inter-feed coupling and has very low cross-polarization. Moreover, a fence made from periodic inter-spaced orthogonal baluns is used to overcome propagating modes. In a similar design technique, a resistive sheet is used in another array design to successfully suppress the in-band resonances, which resulted in more than 13:1 bandwidth [56]. Notably, the frequency selective nature of the resistive sheet is also used to minimize power dissipation. A wide-scan phased array, using frequency selective surface (FSS) with more than 6:1 bandwidth [57]. More than ±60° scan in each plane was achieved using a novel superstrate comprised of printed FSS. The radiating aperture made of dipoles, FSS, and feed line were printed on a vertically oriented printed circuit board (PCB), resulting in a lightweight and low-cost structure.

### 3.2. Array Designs for 5G Applications

The phased array antennas are certain to play a significant role in 5G applications, thanks to the desirable attributes such as higher transmission rate, high gain, and shorter latency. In this section, we are reviewing the recent updates of several existing designs of millimeter-wave phased arrays for 5G applications.

**Figure 5:**
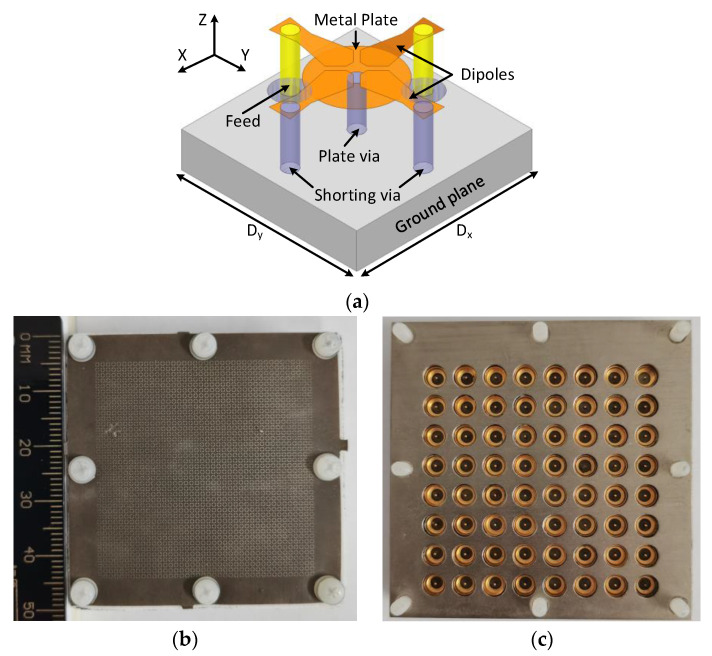
Millimeter-wave array prototype for n257 5G band (**a**) isometric view unit element *D_x_*, *D_y_* = 5 mm; (**b**) top view (including meta-surface loaded WAIM layer); (**c**) bottom view [44].

**Figure 6:**
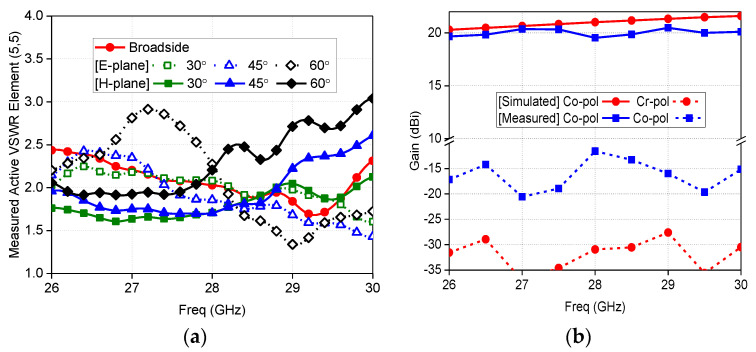
(**a**) 8 × 8 planar 5G millimeter-wave array prototype active VSWR; (**b**) Broadside co-polarized and cross-polarized gain versus frequency [44].

**Figure 7:**
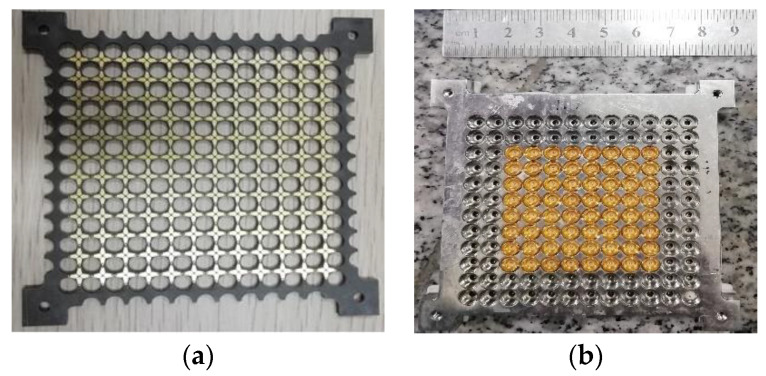
12  ×  12 array prototype designed for 26, 28 GHz 5G millimeter-wave bands (**a**) top view; (**b**) bottom view of the ground plane with 64-element [23].

**Figure 8:**
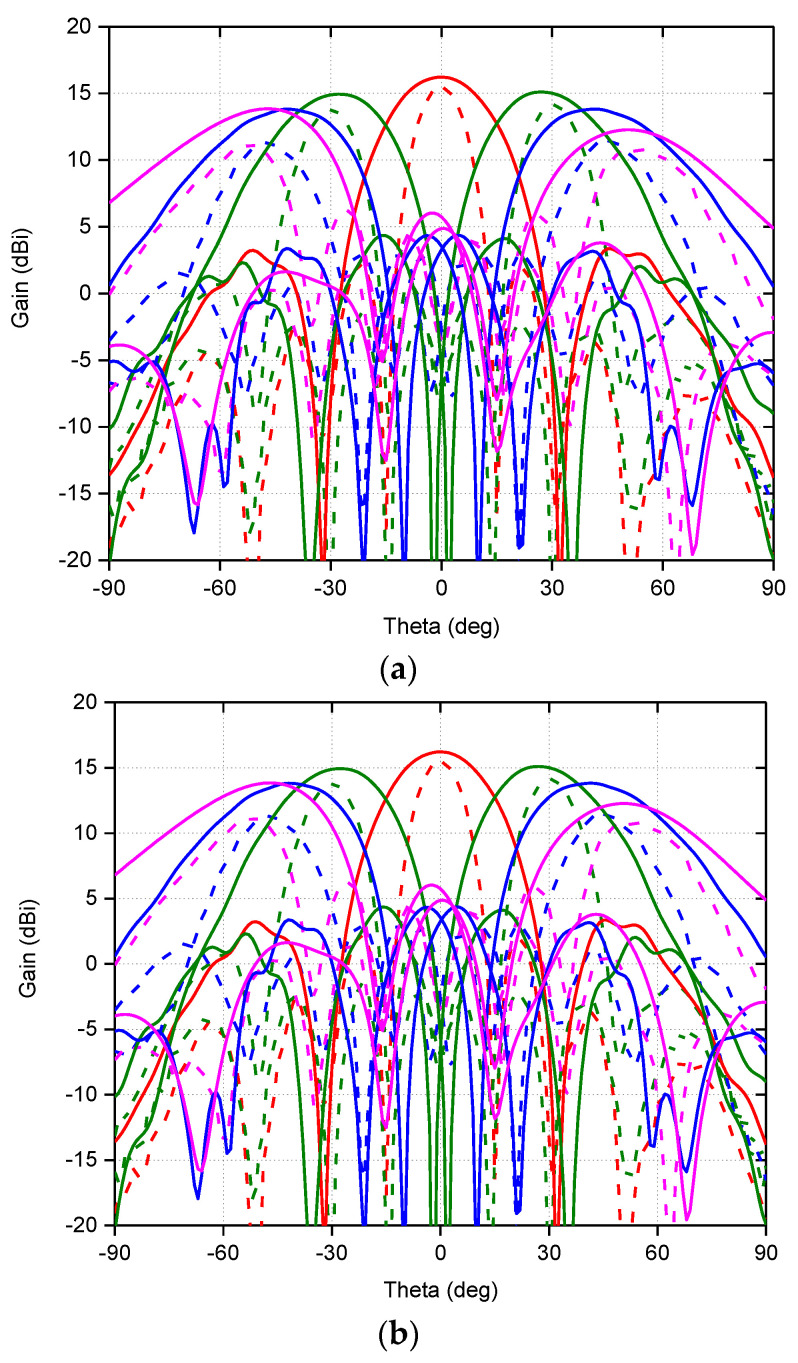
Measured (dashed curve) and simulated (solid curve) scan patterns of 5G millimeter-wave array (32 elements excited) at 26.5 GHz for scan to 0° (red), ±30° (green), ±45° (blue), ±60° (magenta) (**a**) E-plane; (**b**) H-plane [23].

N257 is a very famous 5G band for the initial deployment of technology at millimeter wave-spectrum. This band has a frequency range from 26.5 to 29.5 GHz with a bandwidth of 3 GHz. A low-profile 0.26*λ_h_* dual-polarized meta-surface based array for this 5G is reported [44], the array unit element and fabricated prototype details are presented in Figure 5. This design achieved wide-scan performance with the highest gain of 19.51 dBi at 28 GHz, as illustrated in Figure 6a,b, respectively. Similarly, n257 and n258 (24.25 to 27.5 GHz) bands are covered by [23] 24.25 to 29.50 GHz range with 6 GHz bandwidth exhibiting high gain, efficiency, and good cross-polarization, as the prototype is reproduced in Figure 7 with scan patterns for 26.5 GHz are presented in Figure 8, depicting high gain and low cross-polarization. 

An array consisting of dual-polarized tightly coupled cross-dipoles developed for base station applications is reported [59]. The effects of mutual coupling and its impact on standard performance indices are discussed in detail in this work. The design strategy is based on a novel simplified dipole array model and was proposed due to the lack of regulations standards for 5G, at that time. Moreover, in the author’s view future technology developments can be pursued by investigating the operating principles that govern current base station antennas on 3G/4G platforms. Another base station design used, conducting walls with ferrite sheets and slits are used to design a dual-polarized linear array [60]. This wideband (1–2.83 GHz) and low-profile (0.2*λ_h_*) one-dimensional design used the conducting walls and ferrite sheets to provide PEC boundary to horizontally polarized elements, and also it acted as PMC boundary for vertically polarized elements thus, it seems that elements are located in a planar structure. Green’s function-based equivalent transmission line model, for in-depth analysis of the edge effects in wideband arrays of dipoles, is reported [61]. The proposed equivalent models are convenient and simple solutions to minimize and control the edge effect.

**Figure 9:**
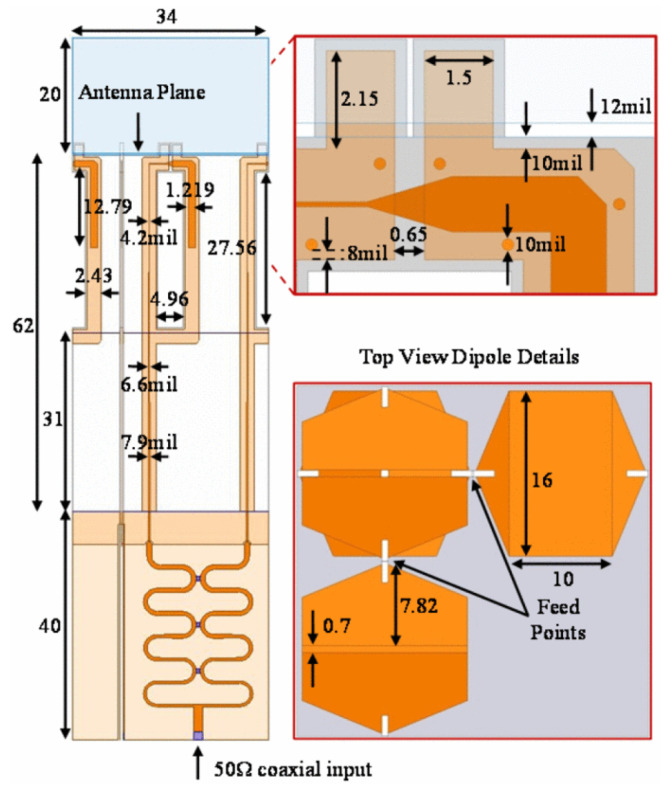
Unit cell illustration. From top to bottom: superstrate, overlapping bowtie elements, resistive sheet, ground plane, and 3-stage Wilkinson power divider [56].

Another 1-D design of tightly coupled dipole array (TCDA) used a vertical parasitic superstrate layer to compensate for the variation in impedance during beam scan [62]. An advantage of this design is its lightweight due to the vertical parasitic superstrate. This linear array achieved 5:1 bandwidth with a wide-scan ±60°. Eight tightly coupled printed dipole units are used to fabricate a linear array for sub 6 GHz 5G bands [63]. This array antenna can be an excellent choice for future multiple-input-multiple-output (MIMO) beamforming base station applications. Parasitic strips are used in this design to achieve a wide scan with a low-profile (0.176λ_2.2 GHz_). Another array design for sub-6 GHz 5G communication is based on tightly coupled patch elements with multiple feeds as reported in [64]. The antenna array design characteristics are studied using a 2-port network model. The size compactness, and lightweight, along with good radiation characteristics, are essential attributes that make this array antenna a suitable candidate for future communication systems like 5G. This design has similar approach as resistive sheet based design whose unit element is shown in Figure 9. Conductive textiles based on tightly coupled arrays with a 30:1 bandwidth are made of three overlapping dipoles [65]. The elements were fed in phase to ensure a uniform current flow. The reported array can be used as a 5G directive wearable antenna in body-worn applications.

A novel cavity-backed array element is used in [66] to achieve wide-scan and metal base parasitic striplines printed on superstrate. The back cavities of the adjacent antenna elements are not isolated but connected to form an array. The array achieved a 60° scan in all azimuth planes. An improved version of PUMA with over 6:1 bandwidth was implemented with an unbalanced feed and without using the external matching network [42]. The shorting via was reconfigured into capacitively-loaded, a modification from the previously reported class of PUMA. Also, bandwidth-limiting loop modes towards lower frequency band end and common-mode resonance were mitigated in this new class. An array suitable for the sub-6 GHz 5G band and other bands with low scattering characteristics is reported in [40]. The scattering performance of the designed array was compared with the tapered slot array (TSA) having the same aperture size, by fabricating two prototypes 6 × 6 proposed array and a 4 × 8 reference array (TSA). The measured radiation performances and scattering results mentioned in this work showed superior monostatic scattering cross-section (SCS) reduction as compared to the reference Vivaldi (TSA) array. A 9:1 dipole array employed tightly coupled topology to 60° scan in all azimuthal planes [67]. A wideband feeding network based on folded Marchand balun was used to achieve impedance transformation. Moreover, instead of a bulky superstrate, FSS is used to achieve wide-scan performance. This approach is somewhat similar to [60] and also achieved a low profile of 0.1*λ*_2*GHz*_. 

**Figure 10:**
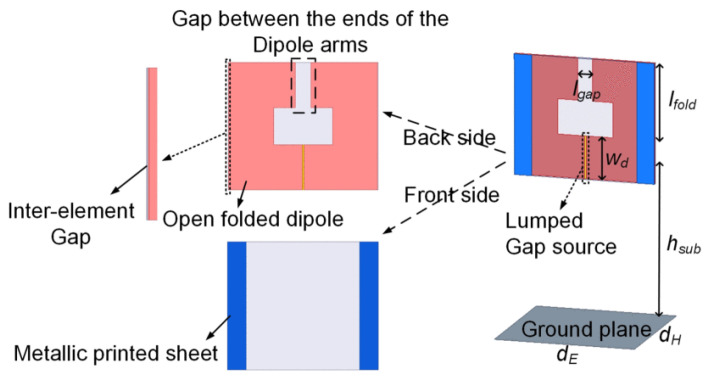
Unit element design of the tightly coupled open folded dipole array [68].

Open folded dipoles are used to achieve UWB impedance matching with a large scan range [68]. The dielectric superstrates inevitably increase the weight of the array, this design is without dielectric superstrates. Compared to the normal diploes in this design, the gap between the open folded dipoles resulted in additional capacitance. This added capacitance resulted in ultra-wideband impedance matching. The 7.33:1 bandwidth along with ±70° achieved by this design are significant considering the design did not use any superstrate, the unit element is shown in Figure 10. In Table 3, the reported CSA designs for various 5G bands are compared in terms of bandwidth, scanning range, unit element dimensions, and gain. Notably, dimensions of the unit element are mentioned in terms of *λ_h_*, i.e., wavelength at the highest operating frequency for a fair comparison. Moreover, all bandwidths are calculated under the criterion of active VSWR ≤ 3.

An interesting array design that successfully integrated with Marchand balun is reported with 10.5:1 bandwidth and wide-angle scanning [69,70]. A fundamental equivalent circuit for CSA is used to design the array by considering the dipole inductance dipole and interelement capacitive coupling to achieve efficient unit element design. A low-profile, tightly coupled array with a simple feed network and 5.5:1 bandwidth is reported in [70]. Tapered Klopfenstein microstrip lines are used to design the feed network, comprising the balun and meandered impedance transformer. This tightly coupled antenna array used Jerusalem cross, printed on both sides of the superstrate as a wide-angle impedance matching layer. Polarization conversion metamaterial is used to preserve the radiation characteristics along with scattering cross-section (SCS) reduction [71]. The design did not use any WAIM layer but still achieved a wide-scan performance in all planes. Significant monostatic SCS reduction is achieved for under normal incidence. The power divider is placed on the ground plane horizontally. The thickness (0.27*λ*_18*GHz*_) acquired by the array without a superstrate matching layer is lower than many reported designs. Conventional dielectric slabs used as WAIM are replaced with novel split rings in [72] to a lightweight array with low-profile height. Used planar split rings contributed to achieving additional reactive components for a wider band. This design is without WAIM, however, notably, the performance of this design is similar to open folded dipoles design without WAIM [68] but with a significantly lower profile.

An integrated balun is used for dual-polarized design including a sub-6 GHz 5G band with cross-located coupled dipoles [67]. The balun served as an impedance transformer that resulted in a wideband feeding network. Sometimes the feeding networks limit the bandwidth of the array, as discussed in the previous section for [53] however, the novelty in this design is the realization of a wideband feed. FSS is used instead of WAIM dialectical slabs for 60° scanning volume in all azimuthal planes. Vertical metal strips are used in linear array design to achieve an impedance bandwidth of more than 3:1 [39]. The impedance matching and beam scanning results of this metal strips-based design are better than [72]. The design gives valuable insights into low-frequency band impedance mismatch by using Floquet theory. A cylindrically conformal array used tightly coupled dipoles in a dual configuration to achieve a 64-element conformal structure [81]. A metallic plate beneath the cross-section of adjacent aperture elements loaded with shorted via added capacitance coupling significantly improved the low-band end performance. This design signified the importance of CSA for conformal applications. A [74] linear millimeter-wave phased array for a mobile terminal used the TCDA technique to achieve 22.5–32.5 GHz bandwidth. The design has a size that is compact enough to be placed at the edges of the mobile. The black-box method is used to realize wide-scan and wideband performance with low scattering characteristics [66]. The proposed black-box method accelerated the monostatic scattering and optimization of radiation of the unit element antenna. The Black-box method is an analysis method commonly used for unknown two-port networks. This approach is handy for designs where an equivalent circuit model is difficult to analyze.

**Figure 11:**
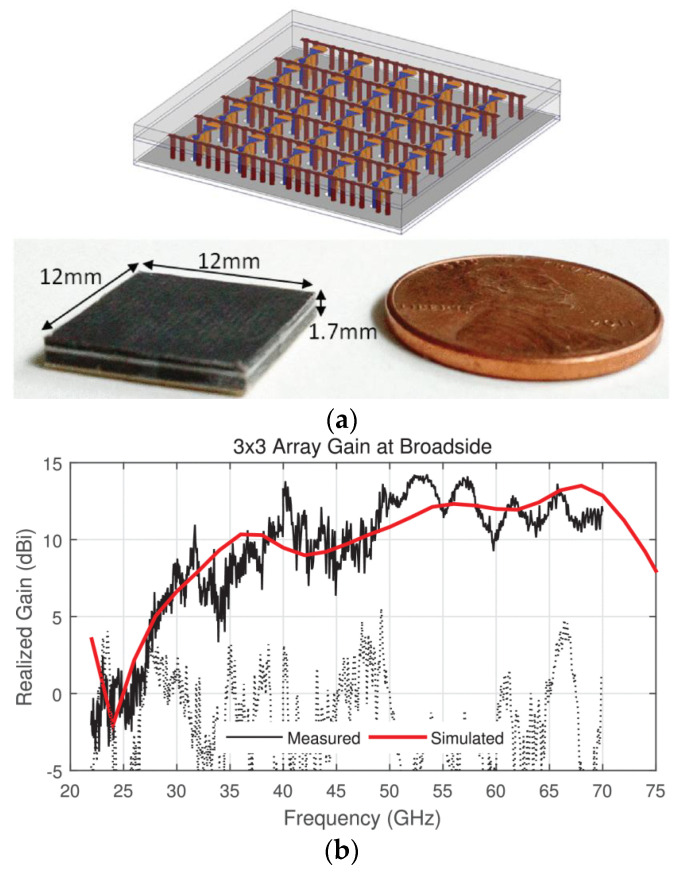
(**a**) 5 × 5 UWB phased array prototype for millimeter-Wave ISM and 5G bands, without measurement fixtures alongside a U.S. penny. Inset (top): array interior structure; (**b**) Broadside gain of the 3 × 3 array prototype. Co-polarized (solid curve) and cross-polarized (dashed) [73].

**Figure 12:**
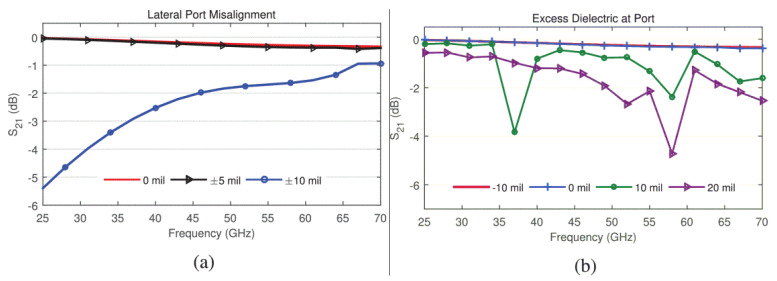
Simulated S_21_ of the coax to microstrip transition, (**a**) coax port shifted laterally by 0–0.254 mm); (**b**) coax port shifted axially by −0.254–0.508 mm [73].

A multiband (24–72 GHz) phased array [73] covered by six 5G millimeter-wave bands is designed as a single printed circuit board. The design deployed H-wall for resonance mitigation due to unbalance feed (Figure 11a). The transmission line model of antenna and balun is considered to access the limitations of fabrication caused by the feed. Prototype fabrication is achieved with an array of elements integrated into a single PCB panel. Due to 72 GHz upper band operation, the prototype fabrication is a challenge because of the minimum achievable feature size, due to the fabrication limitations and requirement of connectors for measurements, the feed and coax footprint are kept identical, and the array is simply shifted to align the appropriate elements, with corresponding S-parameters are reproduced in Figure 12. The planar array resulted in a sharp gain drop at the lowest frequency this is attributed due to the array size (3 × 3) being < λ/2 at the lowest frequency (24 GHz), as dimensions are mentioned in Figure 11. The low-cost PCB fabrication made it ideal for bulk production, with a satisfactory gain performance as shown in Figure 11b. This UWB beamforming array successfully presented the state-of-the-art array for next-generation communication with multifunctional utility.

An array adapted an equilateral triangular lattice to achieve grating-lobe-free scan performance with reduced element count in [43]. By comparing the triangular lattice with a square lattice aperture of similar size, it is established that the triangular configuration demonstrates a large aperture with fewer elements. Also, this work gives valuable insights regarding triangular lattice configuration, very few designs are reported with this configuration in the literature. The tightly coupled design used, the resistive card, and FSS are used to suppress the ground plane interference [82]. Tapered stripline balun is efficiently used for superior impedance matching.

A self-complementary tightly-coupled is used to achieve a wide scan and wideband magnetic current antenna [75]. A reflective cavity is placed beneath the array. This design approach achieved 3:1 bandwidth in sub-6 GHz 5G bands with ±80° and ±70° scan range in E and D-planes, respectively. A tightly coupled dipole array, which is sparsely excited, is designed using a different architecture is reported in [83]. To suppress the grating lobe effect due to the irregular partition, the difference in embedded element pattern is used. This innovative design approach solved the problem of element spacing in CSA, which is usually equal to or less than half of the wavelength at the highest operating frequency. Thus, contributing to avoiding the difficulty in miniaturized connectors, particularly for high-frequency array designs.

**Figure 13:**
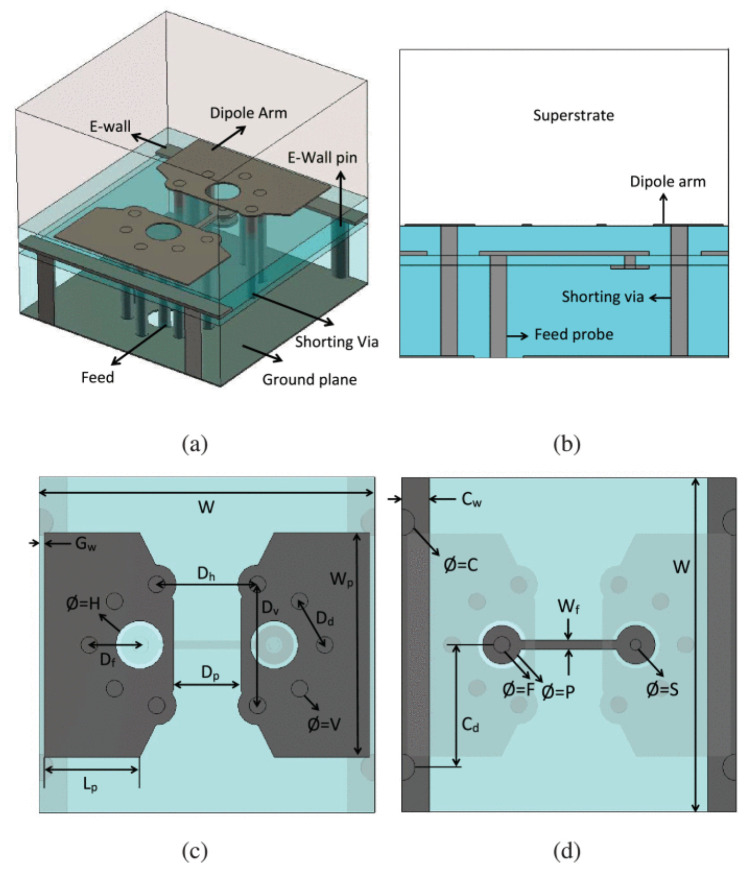
Unit element geometry of fully planar Ultrawideband tightly-coupled array (FPU-TCA), with different views (**a**) 3-D; (**b**) cross-sectional; (**c**) dipole layer; (**d**) horizontal part of the feed probe [76].

**Figure 14:**
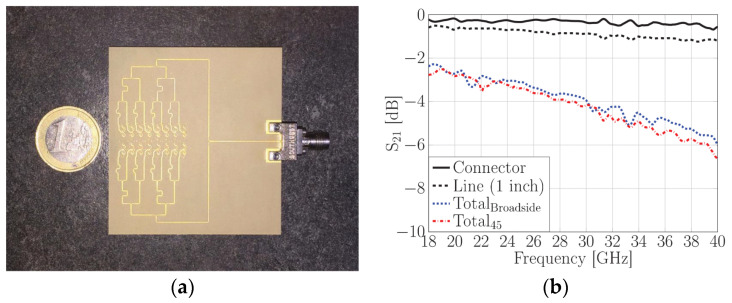
(**a**) Backside view of fully-planar wide-scan millimeter-wave 8 × 4 array prototype in H-structure for scanning to 45°; (**b**) Measured S_21_ of the connector and line and the total value corresponding to the feed networks for the broadside and 45° radiation [76].

**Figure 15:**
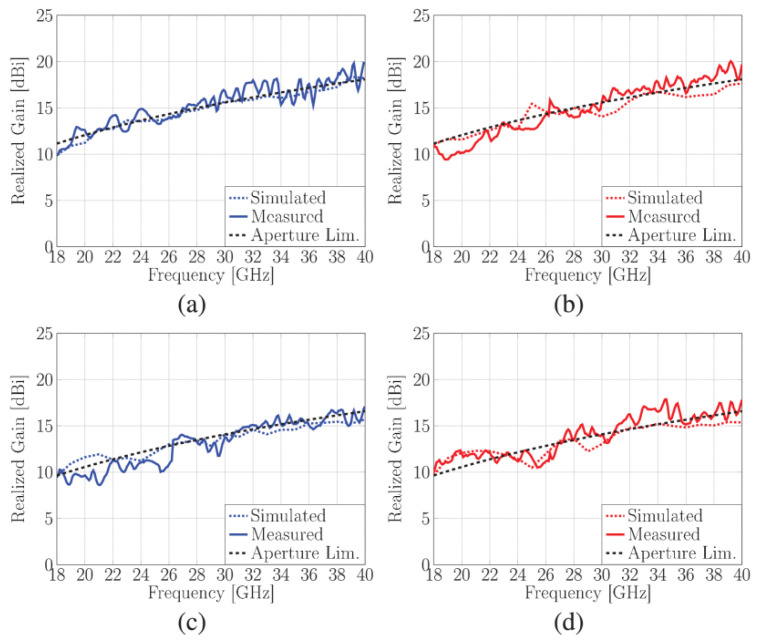
Simulated and measured normalized realized gain of 8 × 4 arrays for (**a**) broadside radiation in E-plane; (**b**) broadside radiation in H-plane; (**c**) 45° radiation in E-plane; (**d**) 45° radiation in H-plane [76].

Another phased array design for millimeter-wave 5G applications with integrated feed has been reported recently [76], unit element of FPU-TCA with different views is elaborated in Figure 13. The feed structure in this design is in reality implemented using a simplified arrangement of plated via holes, which is more cost-effective than the design approach with multi-layer microfabrication. However, higher compared to PCB without blind vias. The prototype (Figure 14) is made of eight columns of four elements. The realized gain and radiation pattern results of longitudinal (along the array axis of eight elements) and the transverse axis (along the array axis of four elements) suggest the high efficiency, of this novel design. This design covered 26, 28 GHz (n257, n258), and 37, 39 GHz (n261) 5G millimeter-wave bands with a wide-angle scan and high gains (Figure 15). An antipodal dipole array with tightly coupled elements and a low cross-polarization feature is designed to operate for more than 5:1 bandwidth [84]. The array elements are fed using a tapered balun with vias placed at the edges of the balun. 

A fully split ring-based meta-surface printed onto the superstrate is used as WAIM to improve the scan volume and low profile. Horizontally polarized dipoles are used to design circular arrays based on the CSA design of tightly coupled elements by placing conducting reflector behind the aperture [85]. The overlapped dipoles are placed above the ground with a capacitive structure at the front, a reflector is placed behind, and a ring of the dielectric slab above to achieve 3.5:1 bandwidth. The designed array has the advantage of using it as a four-sectoral array or omnidirectional array since it acquired a consistent radiation pattern. It is enlightening to inspect radiated fields as a function of scan angle (as mentioned in Table 2 and Table 3). Full-wave simulation predictions of element patterns for infinite arrays may differ from finite array measurements (especially for electrically small arrays) but still are a good indicator of polarization performance. The ring-shaped dielectric slab is effectively used to mitigate the radiation nulls. A polarization-sensitive FSS is used as WAIM in one of the recent array designs with tightly coupled elements and low input resistance with wide-scan volume [86]. The array is fed using microstrip-to-coaxial transition, avoiding an additional impedance transform need.

Instead of the traditional brute-force approach, an equivalent circuit model (ECM) approach is used to design a tightly coupled dipole array with nearly 12:1 bandwidth [87]. The ECM is used to develop the feed and radiating structure, with the model accurately estimating the impedance characteristics. A novel dual-diamond-shaped dipole array is demonstrated for dual-polarization, with compact and wideband feed. A circularly polarized array is reported based on the CSA approach with connected parallel slots for wideband operation [88]. External balun is avoided by using a connected slot that also contributes to low active VSWR. A metal reflector is underneath the aperture to avoid back radiation, and microstrip lines are used as the feeding. A compact UWB array with a single layer feeding network [89] consists of a microstrip-to-slotline structure that significantly reduces the antenna element complexity and size, and is designed for wide-angle scanning capabilities. The low profile of the array is also an added advantage of 0.12*λ_h_*.

**Figure 16:**
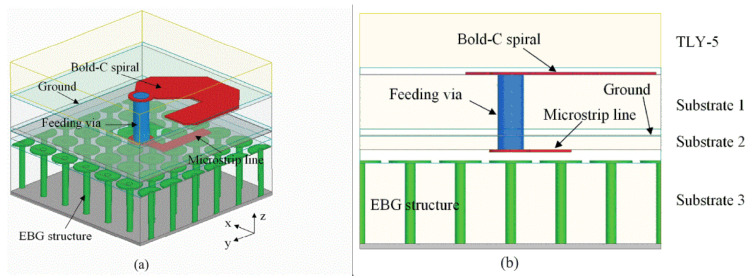
Geometry of the proposed millimeter-wave circularly polarized arrays unit element (**a**) 3-D view; (**b**) Side view [79].

**Figure 17:**
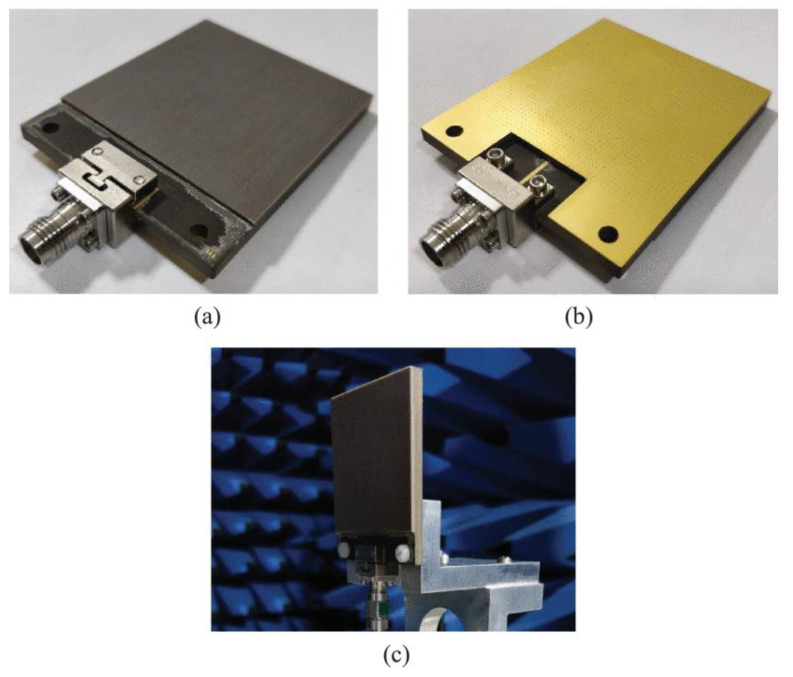
Fabricated prototype, (**a**) top view; (**b**) bottom view; (**c**) testing in the anechoic chamber [79].

**Figure 18:**
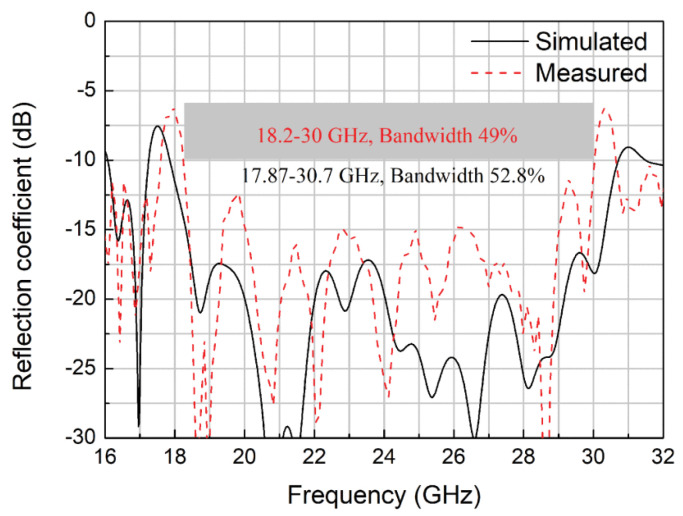
Measured and simulated reflection coefficients of the 8 × 8 CP array antenna for millimeter-wave applications [79].

**Figure 19:**
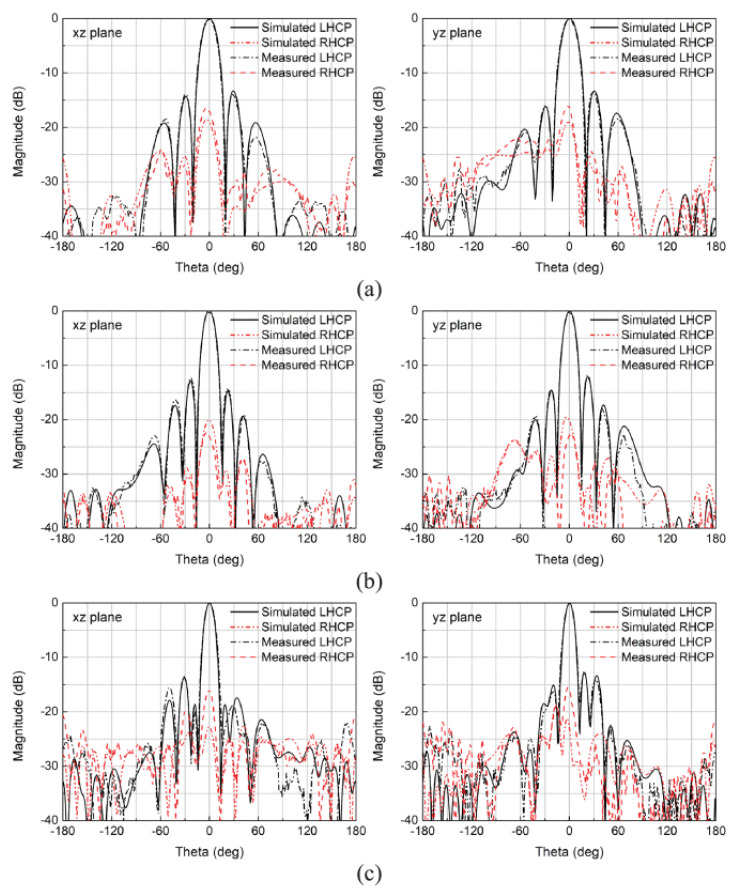
Measured and simulated radiation patterns of 64-element CP millimeter-wave array at (**a**) 19; (**b**) 24.5; (**c**) 30 GHz [79].

**Figure 20:**
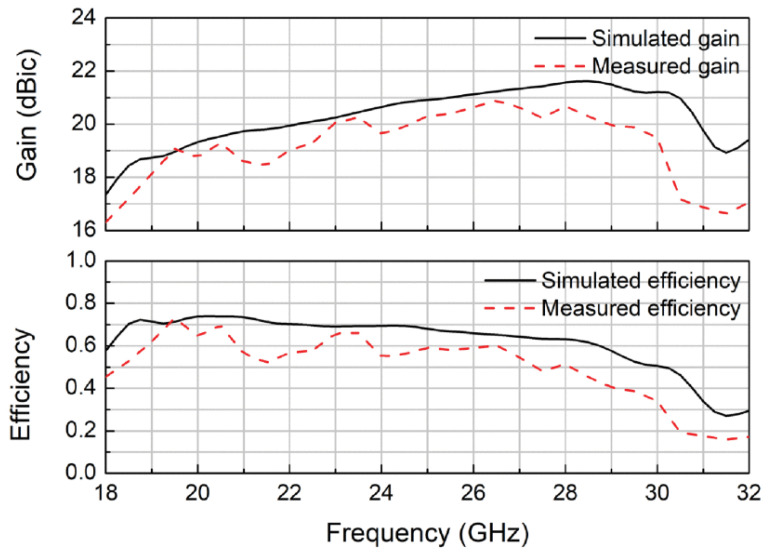
Measured and simulated gains and efficiencies of the CP array designed for 24, 26, 28 GHz 5G bands [79].

A novel approach for bandwidth enhancement achieved by integrating a semi-resistive FSS consisting of band-stop FSS and non-resistive low-pass, in TCDA design achieved 28:1 bandwidth for sub-6 GHz 5G bands [77]. The semi-resistive FSS network suppressed ground plane shorts and preserved the losses towards the upper-frequency band end. A multi-layer metallic strip loaded tightly couple array for smooth impedance transform from aperture to the free space is reported for the sub-6 GHz band [78]. ECM is also proposed for this array with a low-profile and simple feeding structure. Single-arm C-shaped spiral elements are used in a CP array with UWB operation in millimeter 5G bands [79]. As shown in Figure 16, the unit element of the array consists of four stacked substrates, with a thickness of approximately 0.12λ at the center frequency. Dielectric-based inverted microstrip gap waveguide (DIMGW) technology is used for compact feeding network layout (Figure 17). The measured and simulated reflection coefficients agree well with each other for 18–30 GHz millimeter-wave bands, as evident from Figure 18. Moreover, good agreement between the measured and the simulated left-hand circular polarization (LHCP) radiation patterns. Since the designed structure of bold-C elements therefore sidelobe levels are also not symmetric, which is also verified by the measured outcomes (Figure 19). The measured and simulated gains and total efficiency of the array as a function of frequency are shown in Figure 20. The reported maximum measured gain is almost 21 dBi at 26.5 GHz, with measured efficiency nearly above 50% for the majority of the band.

A fully integrated UWB array with a large scan range comprised of asymmetric tightly coupled dipole with a coupling patch beneath dipole to simultaneously eliminate the common-mode resonance and achieve a lower array profile is reported very recently [90]. Three layers of WAIM with different structures are used to achieve more than 60° in principal planes. The coupling patches connected to the ground using the metal wall are used to increase the capacitive coupling between the patch and diploes resulting in bandwidth extension. CSA is integrated with a powerful combination network to realize a low-volume coaxial cable-less array module [91]. This tile-based network architecture achieved a low-volume active power combination network. A multi-functional wideband array deployed a balanced wideband impedance transformer to design a base-station array for mid-band 5G applications [80]. A wideband differential feed with resonance-free scan to 60° and 45° in E-, H-plane is achieved for radio frequency front ends. Thus, fulfilling the multifunctional utility of next-generation communication links.

## 4. The Way Forward

The antenna is a critical component of any wireless communication system and has a significant impact on system performance. Wideband antenna arrays are ideal to achieve multi-functionality, large channel capacity, and high data rate. The overall system cost can be decreased if the radio front-end supports multiple band communication. Although a great deal of work has been done to produce wide-angle and wideband phased arrays, technology demands change quickly. By reducing second-order harmonics, modern digital phased array radios and differential RF front ends improve distortion and noise immunity. The latest RF front-end innovations offer good linearity, low noise, and high dynamic range in the transceiver chain. However, there are still several essential roadblocks to overcome.

Antenna-on-chip arrays are compact and integrate the array directly above the transceiver circuitry; however, low efficiency due to silicon substrate is a big drawback. The flip-chip interface is used for array antenna-in-package by utilizing a low-loss substrate and is commonly realized using PCB technology. However, these approaches still support narrowband applications. For 5G systems and beyond that demand wideband performance, an essential requirement is an antenna array that can be efficiently realized in a single step, with low complexity and cost. 

As we move to millimeter-wave frequencies, scalability and manufacturing feasibility are essential considerations. For commercial production, assembly complications and fabrication costs are of paramount importance. Thus, the significant challenge on the way ahead is the planarization and simplification of millimeter-wave antenna arrays to achieve low-cost, high efficiency, ultra-wideband, and wide-scan features.

Spectrally agile phased array antennas are also one of the future trends. Antenna arrays that will be scalable in size and frequency can efficiently meet the fast-changing technology demands of future communication and sensor systems. Therefore, viable future antenna array technologies should be adaptable and scalable to meet the needs of an ever-evolving next-generation communications architecture.

## Figures and Tables

**Table 1:** Multi-layer spectrum for 5G to address a wide range of requirements.

Layers	Key Characteristics	Spectrum
Coverage and capacity	Primary band for 5G intro Decent coverage and capacity	Sub-6 GHz 3.3–4.2 GHz 4.4–5 GHz
Super data	Extremely high data rates Limited coverage	Millimeter-wave 24–29 GHz, 37–42 GHz 57–64 GHz, and above
Coverage	Wide-area coverage Deep indoor coverage	Below 2 GHz e.g., 700 MHz

**Table 2:** A comparison within the reported CSA references with UWB operation.

Ref.	Bandwidth	Max Scan Angle (E-, H-, D-Plane)	Unit Element Dimensions (*λ_h_*)	Maximum Realized Gain (dBi)	Insights/Salient Features
[38]	7:1 (0.68–5.0 GHz)	45°, 45°, 45°	0.24 × 0.24× 0.68	19	Marchand balun reactance is used for impedance matching A wideband balun non-bulky balun led to size, weight, and cost reduction
[41]	5:1 (1.06–5.3 GHz)	45°, 45°, 45°	0.43 × 0.43 × 0.46	NA	Feeding method which eliminated the external balun Common-mode resonance mitigation using shorting posts
[47]	21:1 (0.28–5.91 GHz)	NA *	0.46 × 0.46 × 1.15	17.5	FSS incorporated with tightly coupled elements FSS suppresses destructive ground plane interference, to achieve wideband with low-profile
[48]	20:1 (0.85–17 GHz)	NA	NA × NA × 0.43	NA	Interwoven arms to achieve wideband Equivalent circuits are proposed to model the radiation impedance
[52]	4:1 (170–700 MHz)	NA, 30°, NA	0.7 × 0.7 × 0.25	12.8	Edge effect alleviation using short-circuit terminations of periphery elements Proposed termination provides a good tradeoff between impedance bandwidth and realized gain
[54]	4.5:1 (5.6–25.5 GHz)	40°, 40°, 40°	0.59 × 0.59 × 0.71	NA	An efficient circuit model to optimize the design of connected as well as tightly coupled dipole arrays The circuit model considers the ground plane and dielectric layers
[55]	6:1 (0.6–3.6 GHz)	60°,60°, NA	0.41 × 0.41 × 1.04	23	Fence of the periodic inter-spaced orthogonal balun to overcome propagating modes Split unit cell with minimized inter-feed coupling
[56]	13:1 (290–3900 MHz)	45°, 45°, 45°	0.44 × 0.44 × 1.59	20	A resistive sheet is used to suppress the in-band resonances A dielectric layer is deployed as a superstrate to maintain high efficiency
[57]	6:1 (0.5–3.1 GHz)	75°, 60°, 70°	0.50 × 0.50 × 0.74	23	Superstrate comprised of printed FSS Replacement of bulky superstrate layers with periodic printed elements and yet achieve wide-angle and wideband impedance matching

* NA means not mentioned in the reference.

**Table 3:** A comparison within the reported CSA references for various 5G applications.

Ref.	Bandwidth/5G Band	Max Scan Angle (E-, H-, D-Plane)	Unit Element Dimensions (*λ_h_*)	Maximum Realized Gain (dBi)	Insights/Salient Features
[23]	23.5–29.5 GHz/26, 28 GHz	60°, 60°, 60°, Dual-polarized	0.53 × 0.53 × 0.37	19.5	Dual-polarized structure with sub-wavelength split-ring resonators loaded WAIM for wide-scan volume Metal plated loaded shorting for better capacitance and resonance mitigation
[63]	1.91–5.35 GHz/Sub-6 GHz	NA *	0.37 × 0.44 × 0.22	11.2	Wide bandwidth operation of rectangle patches in the tightly coupled environment Compact size, lightweight, broad bandwidth, and good radiation characteristics
[64]	2.2–6 GHz/Sub-6 GHz	45°, -, -	0.8 × 0.48 **	> 6.9	Coplanar waveguide with ground-feeding network Two rows of parasitic strips as WAIM to achieve wide scan
[68]	0.3–2.2 GHz/Sub-6 GHz	70°, 50°, 50°	0.36 × 0.36 × 0.69	19	The tightly coupled elements are open folded dipoles without additional dielectric superstrates Gaps due to open folded structure provided additional capacitance, thus resulting in better impedance matching
[70]	0.80–4.38/Sub-6 GHz	70°, 55°, -	0.35 × 0.35 × 0.48	22	The feed network with meandered impedance transformer and Klopfenstein tapered microstrip lines-based balun The wide-angle impedance matching using a novel wideband Jerusalem cross based metasurface superstrate
[72]	0.3–2.15 GHz/Sub-6 GHz	70°, 45°, -, Dual-polarized	0.5 × 0.5 × 0.48	18	Novel split ring to replace the conventional WAIM superstrates Low profile, lightweight, and excellent polarization purity
[73]	24–72 GHz/24, 26, 28, 38, 60, 66 GHz	45°, 45°, 45°	0.5× 0.5 × 0.39	14	Continuous coverage of all six 5G bands Inherent planar array co-fabricated as a single PCB for significant cost reduction
[74]	22.5–32.5 GHz/24, 26, 28 GHz	30°, -, -	0.52 × 1.3 **	10.5	Compact design to install on the edge of mobile terminals One-to-two Wilkinson power divider as a balun
[75]	1.85–6.05 GHz/Sub-6 GHz	80°, 40°, 70°	0.41 × 0.41 × 0.12	23	Self-complementary design to achieve wide-scan performance in E-plane WAIM for better impedance matching and large scanning angle
[76]	17–42 GHz/24, 26, 28, 37 GHz	45°, 45°, 45°	0.42 × 0.42 × 0.43	20	TCDA with an integrated feed network with the configuration of plated via holes Common-mode suppression using H-wall
[77]	0.20–5.6 GHz/Sub-6 GHz	60°, 45°, 45°	0.47 × 0.47 × 2.05	13	Bandwidth enhancement by introducing a semi-resistive FSS Semi-resistive FSS network to suppresses ground plane shorts
[78]	1.4–8.68 GHz/Sub-6 GHz	60°, 60°, 60°	0.47 × 0.47 × 0.8	19	Multilayer metallic strips to achieve smooth impedance transformation from dipole to the free space All components of the array are printed on a single substrate, leading to low-cost and lightweight
[79]	18.1–30.4 GHz/26, 28 GHz	NA, circular polarization	0.57 × 0.57 × 0.44	> 21	Single-arm C-shaped spiral elements for CP tightly coupled array DIMGW technology for compact layout feed layout
[80]	1.05–3.20 GHz/Sub-6 GHz	60°, 45°, 45°	0.50 × 0.50 × 0.24	NA	Novel differential feed based on balanced wideband impedance transformer A pair of reactive transmission line components are employed to mitigate common modes

* NA means not mentioned in the reference, ** 1-D array.

## Data Availability

Not applicable.
